# Emerging Therapeutic Strategies for Fragile X Syndrome: Q&A

**DOI:** 10.1021/acschemneuro.2c00674

**Published:** 2022-12-07

**Authors:** Ghassan Alusi, Elizabeth Berry-Kravis, David Nelson, Lauren L. Orefice, Sam A. Booker

**Affiliations:** † Barts Health NHS Trust, LondonE1 2ES, U.K.; ‡ Department of Pediatrics, Neurological Sciences, and Anatomy and Cell Biology, Rush University Medical Center, Chicago, Illinois60612, United States; § Molecular and Human Genetics, Baylor College of Medicine, Houston, Texas77030, United States; ∥ Department of Molecular Biology, Massachusetts General Hospital, Boston, Massachusetts02114, United States; ⊥ Department of Genetics, Harvard Medical School, Boston, Massachusetts02115, United States; # Simons Initiative for the Developing Brain, University of Edinburgh, EdinburghEH8 9XD, U.K.

**Keywords:** Fragile X syndrome, neurodevelopmental conditions, fragile X messenger ribonucleoprotein 1, arbaclofen, phosphodiesterase, somatosensation

## Abstract

Understanding how best to treat aspects of Fragile X syndrome has the potential to improve the quality of life of affected individuals. Such an effective therapy has, as yet, remained elusive. In this article, we ask those researching or affected by Fragile X syndrome their views on the current state of research and from where they feel the most likely therapy may emerge.

Summer 2022 saw the hotly anticipated (in-person) return of a well-known biennial conference and symposium that focuses on Fragile X syndrome and autism research, in the sunny Italian countryside. The last occasion that many of the eminent researchers in this field had to meet face-to-face in 2018 was (to many) a distant cherished memory that had spurred a new vigor in helping the lives of individuals with Fragile X syndrome. This most recent meeting did not fail to inspire both established researchers and a new cadre of early career researchers. Indeed, the breadth of research being undertaken to understand molecular, cellular, and circuit mechanisms that lead to the range of difficulties experienced by fragile X individuals is giving us greater insight than even before as to how such features emerge during neurodevelopment.

Fragile X syndrome is an X-linked condition that affects 1:(4000–6000) people worldwide. It results from hypermethylation of the *FMR1* (Fragile X messenger ribonucleoprotein 1) gene leading to function loss of the protein product FMRP. Affected individuals often experience altered sensory reactivity, autism, cognitive impairment, epilepsy, and attentional issues; which can necessitate care and treatment throughout life. The importance of basic research in identifying novel targets for treatment, or refining previously identified targets, should not be underplayed as we still have a great deal to learn about how FMRP influences brain function.

Nevertheless, despite great effort in understanding the cell biology of Fragile X syndrome, no therapy has yet been licensed. This is despite notable clinical trials, including arbaclofen (STX-209), a GABA_B_ receptor agonist;^1^ fenobam, mavoglurant, or basimglurant, mGluR5 negative allosteric modulators;^2−4^ metformin, an AMPK modulator;^5^ and many others such as acamprosate, lovastatin, minocycline, metadoxine, trofinetide. While several of these compounds have shown promise, the reasons for the failure of clinical trials are complex.^6^ Beyond pharmacology, there have been attempts to develop gene therapies to re-express FMRP, which are still at preclinical stage.^7^ To ascertain the current state of research and thoughts on future therapeutic strategies, Sam A. Booker posed the following question to leading Fragile X syndrome researchers and relatives of affected individuals:

“Many therapies for Fragile X syndrome have shown great promise in the laboratory but have not made it to the clinic. What do you think are the current promising therapeutic approaches, and where do you think the next advance will come from?”

## Ghassan Alusi, Barts NHS Trust London and Fragile X Family Member, U.K.

I have always maintained that the scientists who are investigating Fragile X syndrome should remain objective, follow rigorous scientific guidelines, and follow the trodden path of preclinical to clinical therapeutics development. The heartbreak of the failure of the arbaclofen clinical trial was particularly difficult for the community of Fragile X families who put their hopes on this trial. Was the preclinical work on rodents and other animals valid? Yes it was. Were the outcome measures specified adequate or the duration of therapy long enough to see an effect? These are questions that remain unanswered so many years on from the trial.

Malaria vaccine development has taken 35 years from the day the antigen was discovered to reach the clinic. Millions of lives were lost in the meantime. The obvious comparison to the rapid development of the COVID vaccine will frustratingly fall on deaf ears and quickly overlooked.

Whether the best therapeutic approach is gene therapy or targeted molecule development is best is not the point. The speed of progress in getting therapies to the clinic for Fragile X syndrome is painfully slow for us as parents, especially when a COVID vaccine was injected into individuals within a year of the start of the outbreak.

Knowing what the scientific and regulatory communities can do in such a short period of time has changed my mind. Let us have more emotional involvement. Let us relax regulatory restrictions, and let us accelerate treatment development. I understand the caution of big pharma when it comes to pediatric therapies especially after previous failures, but I speak for most of the parents of Fragile X syndrome: we are desperate for treatments. These kids deserve a chance at reaching their potential in life.

## Elizabeth Berry-Kravis, Rush University Medical Center, U.S.

I think there is a strong confluence of data building in preclinical studies for phosphodiesterase inhibitors that work on the reduced cyclic AMP production seen in animal models and people with FXS, as well as preclinical evidence that agents acting on ion channels regulated by FMRP may provide good therapeutic benefit. There is also early clinical evidence of a benefit for a phosphodiesterase 4D inhibitor that acts mainly in the brain. These categories of molecules offer great promise, particularly with the improved trials designs and outcome measures that have been developed over the past 5–10 years for FXS, spurred by the early “failed” trials that may not have shown benefit because of problems with outcome measurement and design.

More long-term advances will be combining agents that work on FMRP mechanisms to give additive therapeutic benefit, and use of gene therapy and other genetic strategies, which are in development, to allow production of FMRP in brain and correct the primary defect in FXS. Combinations of genetic and pharmacologic strategies targeted to the underlying disease will likely provide the approach to achieve the most benefit in terms of improvement in the underlying disease in FXS.

## David Nelson, Baylor College of Medicine, U.S.

The field has made significant progress understanding the many roles of *FMR1* and the consequences of its absence. Unfortunately, because *FMR1* interacts with so many target mRNAs, it helps regulate many pathways. Modulating one or a few of these has had beneficial effects in model systems, but translation to humans has not shown similar levels of efficacy. Drugs meant to alter pathways, such as those altering translation of proteins in response to synaptic signals, have worked well in mice and rats; clinical trials have not shown benefit in people. Some drugs remain in the clinical testing pipeline and have shown promise. An example is BPN14770, Tetra Therapeutics’ PDE4D inhibitor that targets specific components of the signal transduction pathway leading to *FMR1* control of mRNA translation. It has shown early success, and larger clinical trials are underway. Another approach has been to recognize the imbalance of excitatory/inhibitory inputs to neurons and to stimulate inhibition with GABA-agonists to help normalize the overstimulation found in Fragile X patients and models. One such drug, arbaclofen, has shown some efficacy in a subset of Fragile X individuals in a large clinical trial. Repetition of this result may help define the characteristics of those who show benefit. Finally, gene replacement therapy may provide the best approach for providing an effective therapeutic strategy. Replacing *FMR1* at normal levels and in the appropriate cells can restore functions of all the pathways it touches, eliminating the need to find drugs to alter each. It is a huge challenge but a worthwhile one. While there may be developmental functions that cannot be replaced in older individuals, based on studies in models, it is likely that restoration of *FMR1* to normal levels will improve many features of the disorder. Recent advances in gene replacement methods have given new enthusiasm for treatment in Fragile X and many other genetic disorders.

## Lauren L. Orefice, Massachusetts General Hospital & Harvard Medical School, U.S.

Our work has focused on understanding somatosensory abnormalities in Fragile X syndrome and autism spectrum disorder (ASD). In our studies, we found that loss of different ASD-associated genes, only in peripheral sensory neurons, is sufficient to cause touch hypersensitivity in mice.^8^ We deleted these ASD-associated genes in peripheral sensory neurons while keeping them intact in the rest of the body and the rest of the nervous system. Surprisingly, this deletion only in the peripheral somatosensory neurons led to major tactile over-reactivity that recapitulated the over-reactivity observed when ASD-associated genes were deleted throughout the nervous system. We also found that touch hypersensitivity, due to dysfunction of peripheral sensory neurons, causes abnormalities in region-specific brain development and leads to increased anxiety-like behaviors and social impairments in several mouse models for autism. This has led us to develop a model in which autism-associated gene mutations disrupt the function of peripheral sensory neurons, which leads to altered and elevated sensory inputs coming into the central nervous system that can result in sensory over-reactivity and related behavioral changes such as altered social behaviors.

These findings have inspired an idea for novel ways to treat sensory issues in individuals with Fragile X syndrome and ASD. Our studies are aimed at producing peripherally restricted compounds that target peripheral sensory neuron excitability as a tractable and simple therapeutic approach. We have shown that these approaches can reduce touch hypersensitivity, and related phenotypes in mice including anxiety-like behaviors and social impairments. Importantly, these therapeutic approaches target the peripheral nervous system and do not need to cross the blood–brain barrier, which limits potential side effects. We are excited about this method, as peripheral sensory neurons are highly accessible and modulating peripheral sensory neuron excitability is a simpler approach than targeting precise neural signaling pathways in specific brain regions. This method using peripherally restricted compounds is also likely to have far fewer side effects than brain penetrating drugs, which is a critical issue if we want to consider treating children with sensory over-reactivity issues.

## Concluding Remarks

In response to the question posed, all responders confirmed the unmet therapeutic need of individuals with Fragile X syndrome. While there is still much to learn about the role of FMRP in individuals and model systems, there is great motivation from researchers and families to work together for the benefit of affected individuals. While gene therapy for Fragile X syndrome is still in its infancy, recent successes in other single gene conditions (such as spinal muscular atrophy) are setting the ground rules for how such therapies may be employed and what hurdles they need to overcome.

One promising candidate treatment discussed by responders has recently shown safety and efficacy in Fragile X individuals, BPN-14,770 (a phosphodiesterase 4D inhibitor),^9^ which is yet to enter phase 3 trials but improved several aspects of life including language and daily functioning in adults during phase 2 trials. Defining how this drug or other compounds previously in clinical trials improve quality of life for individuals over development into adulthood is a priority that has the potential to produce rapid benefits. How these treatments and future approaches unfold over the two years before the next meeting will be hugely important to all.

## References

[ref1] Berry-Kravis E.; Hagerman R.; Visootsak J.; Budimirovic D.; Kaufmann W. E.; Cherubini M.; Zarevics P.; Walton-Bowen K.; Wang P.; Bear M. F.; Carpenter R. L. Arbaclofen in fragile X syndrome: results of phase 3 trials. J. Neurodev. Disord. 2017, 9 (1), 3 10.1186/s11689-016-9181-6. 28616094 PMC5467054

[ref2] Berry-Kravis E.; Hessl D.; Coffey S.; Hervey C.; Schneider A.; Yuhas J.; Hutchison J.; Snape M.; Tranfaglia M.; Nguyen D. V.; Hagerman R. A pilot open label, single dose trial of fenobam in adults with fragile X syndrome. J. Med. Genet. 2009, 46 (4), 266–271. 10.1136/jmg.2008.063701. 19126569 PMC2658751

[ref3] Youssef E. A.; Berry-Kravis E.; Czech C.; Hagerman R. J.; Hessl D.; Wong C. Y.; Rabbia M.; Deptula D.; John A.; Kinch R.; Drewitt P.; Lindemann L.; Marcinowski M.; Langland R.; Horn C.; Fontoura P.; Santarelli L.; Quiroz J. A.; Effect of the mGluR5-NAM Basimglurant on Behavior in Adolescents and Adults with Fragile X Syndrome in a Randomized, Double-Blind, Placebo-Controlled Trial: FragXis Phase 2 Results. Neuropsychopharmacology 2018, 43 (3), 503–512. 10.1038/npp.2017.177. 28816242 PMC5770759

[ref4] Berry-Kravis E.; Des Portes V.; Hagerman R.; Jacquemont S.; Charles P.; Visootsak J.; Brinkman M.; Rerat K.; Koumaras B.; Zhu L.; Barth G. M.; Jaecklin T.; Apostol G.; von Raison F. Mavoglurant in fragile X syndrome: Results of two randomized, double-blind, placebo-controlled trials. Sci. Transl. Med. 2016, 8 (321), 321ra5–321ra5. 10.1126/scitranslmed.aab4109. 26764156

[ref5] Proteau-Lemieux M.; Lacroix A.; Galarneau L.; Corbin F.; Lepage J.-F.; Çaku A. The safety and efficacy of metformin in fragile X syndrome: An open-label study. Progress in Neuro-Psychopharmacology and Biological Psychiatry 2021, 110, 110307 10.1016/j.pnpbp.2021.110307. 33757860

[ref6] Budimirovic D. B.; Berry-Kravis E.; Erickson C. A.; Hall S. S.; Hessl D.; Reiss A. L.; King M. K.; Abbeduto L.; Kaufmann W. E. Updated report on tools to measure outcomes of clinical trials in fragile X syndrome. J. Neurodev. Disord. 2017, 9 (1), 14 10.1186/s11689-017-9193-x. 28616097 PMC5467057

[ref7] Hooper A. W.; Wong H.; Niibori Y.; Abdoli R.; Karumuthil-Melethil S.; Qiao C.; Danos O.; Bruder J. T.; Hampson D. R. Gene therapy using an ortholog of human fragile X mental retardation protein partially rescues behavioral abnormalities and EEG activity. Molecular Therapy-Methods & Clinical Development 2021, 22, 196–209. 10.1016/j.omtm.2021.06.013. 34485605 PMC8399347

[ref8] Orefice L. L.; Mosko J. R.; Morency D. T.; Wells M. F.; Tasnim A.; Mozeika S. M.; Ye M.; Chirila A. M.; Emanuel A. J.; Rankin G.; Fame R. M.; Lehtinen M. K.; Feng G.; Ginty D. D. Targeting peripheral somatosensory neurons to improve tactile-related phenotypes in ASD models. Cell 2019, 178 (4), 867–886. 10.1016/j.cell.2019.07.024. 31398341 PMC6704376

[ref9] Berry-Kravis E. M.; Harnett M. D.; Reines S. A.; Reese M. A.; Ethridge L. E.; Outterson A. H.; Michalak C.; Furman J.; Gurney M. E. Inhibition of phosphodiesterase-4D in adults with fragile X syndrome: a randomized, placebo-controlled, phase 2 clinical trial. Nature Medicine 2021, 27 (5), 862–870. 10.1038/s41591-021-01321-w. 33927413

